# Retraction: Circulating FoxP3+ regulatory T and interleukin17-producing Th17 cells actively influence HBV clearance in *de novo* Hepatitis B virus infected patients after orthotopic liver transplantation

**DOI:** 10.1371/journal.pone.0226148

**Published:** 2019-12-02

**Authors:** The PLOS ONE Editors

Concerns have been raised that the transplants performed in the local context at the time of procedures reported in this article [1] may have involved organs/tissues procured from prisoners [2]. International ethics standards call for transparency in organ donor and transplantation programs and clear informed consent procedures including considerations to ensure that donors are not subject to coercion.

The Ethics Statement in this article [1] states, “None of the transplant donors were from a vulnerable population or were subject to coercion.” The authors provided ethics approval documentation and informed consent forms for organ donors when requested by the journal. The first author commented that this was a retrospective study, that the authors were not involved with organ donation or surgical procedures, and that organ donations were conducted in compliance with local regulations. The first author also claimed that transplant donors did not include a vulnerable population. However, the authors did not provide any further information about donors, donors’ causes of death, or the institutions where donations were secured. Based on the outcome of our follow-up discussions, it remains unclear whether any donor organs for transplant cases discussed in [1] were procured from prisoners.

Furthermore, the ethics approval document provided during follow-up discussions indicated that written informed consent forms were not considered in the ethics review for this study and appeared to be dated after the completion of the study. Per the journal’s policy, ethics approval must be obtained before the beginning of a study involving human subjects or sensitive data (including medical records) from human subjects.

The authors did not provide the primary data underlying their article and as such the article does not comply with the PLOS Data Availability Policy.

Owing to the above concerns, and in line with international ethics standards for organ/tissue donation and transplantation, the *PLOS ONE* Editors retract this article.

The authors did not respond to the retraction notification.

## References

[1] Gao Y, Zhang M, Li J, Yang M, Liu Y, Guo X, et al (2015) Circulating FoxP3+ Regulatory T and Interleukin17-Producing Th17 Cells Actively Influence HBV Clearance in *De Novo* Hepatitis B Virus Infected Patients after Orthotopic Liver Transplantation. PLoS ONE 10(9): e0137881 10.1371/journal.pone.0137881 26367459 PMC4569371

[2] Rogers W, Robertson MP, Ballantyne A, et al Compliance with ethical standards in the reporting of donor sources and ethics review in peer-reviewed publications involving organ transplantation in China: a scoping review BMJ Open 2019;9:e024473 10.1136/bmjopen-2018-024473 30723071 PMC6377532

